# Supplementary material for: PTHrP Regulates Fatty Acid Metabolism via Novel lncRNA in Breast Cancer Initiation and Progression Models
Source: Cancers (Basel). 2023 Jul 25;15(15):3763. doi: 10.3390/cancers15153763 (PMC10417726; doi:10.3390/cancers15153763)
Supplement: Supplementary file 1 [file cancers-15-03763-s001.zip › Figures S1 to S8.pdf]

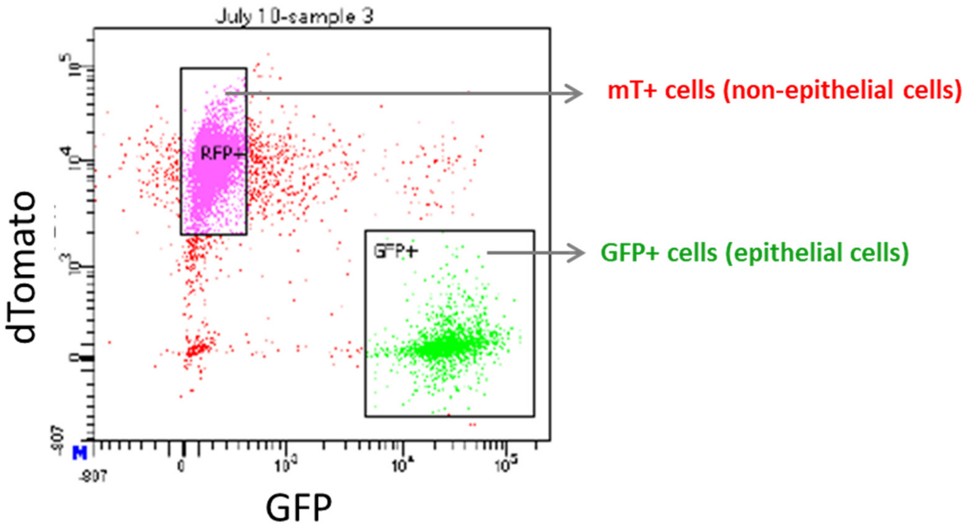

Supplementary Figure S1. Representative FACS of mammary gland cells showing presence of mGFP+ cells in normal mammary gland from Pthrp$^{wt/wt}$; Cre+ mTmG control mice. Shown are Td on the y-axis and GFP in the x-axis.

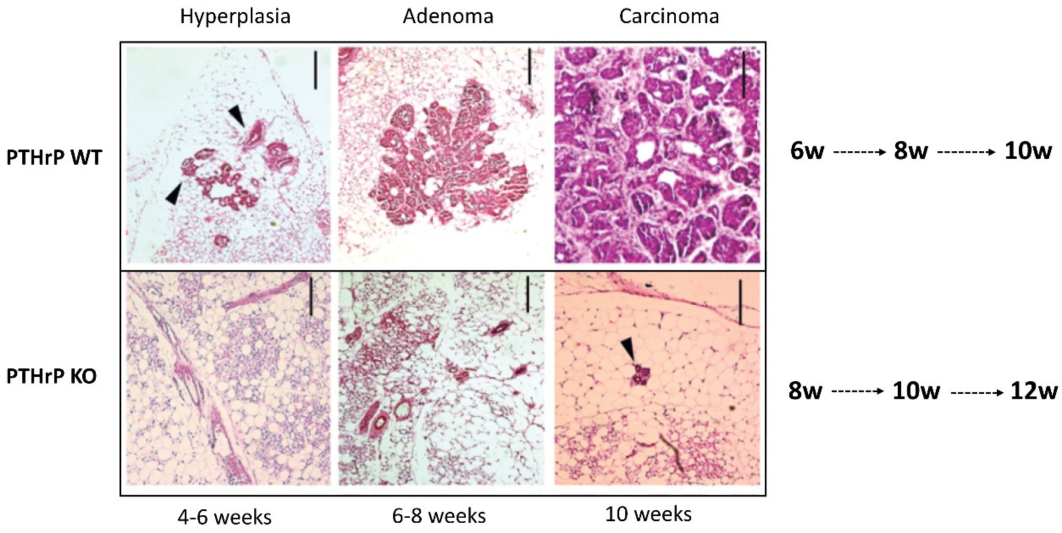

Supplementary Figure S2. RNAseq samples collection from MMTV-PyMT mouse model of 4 groups.

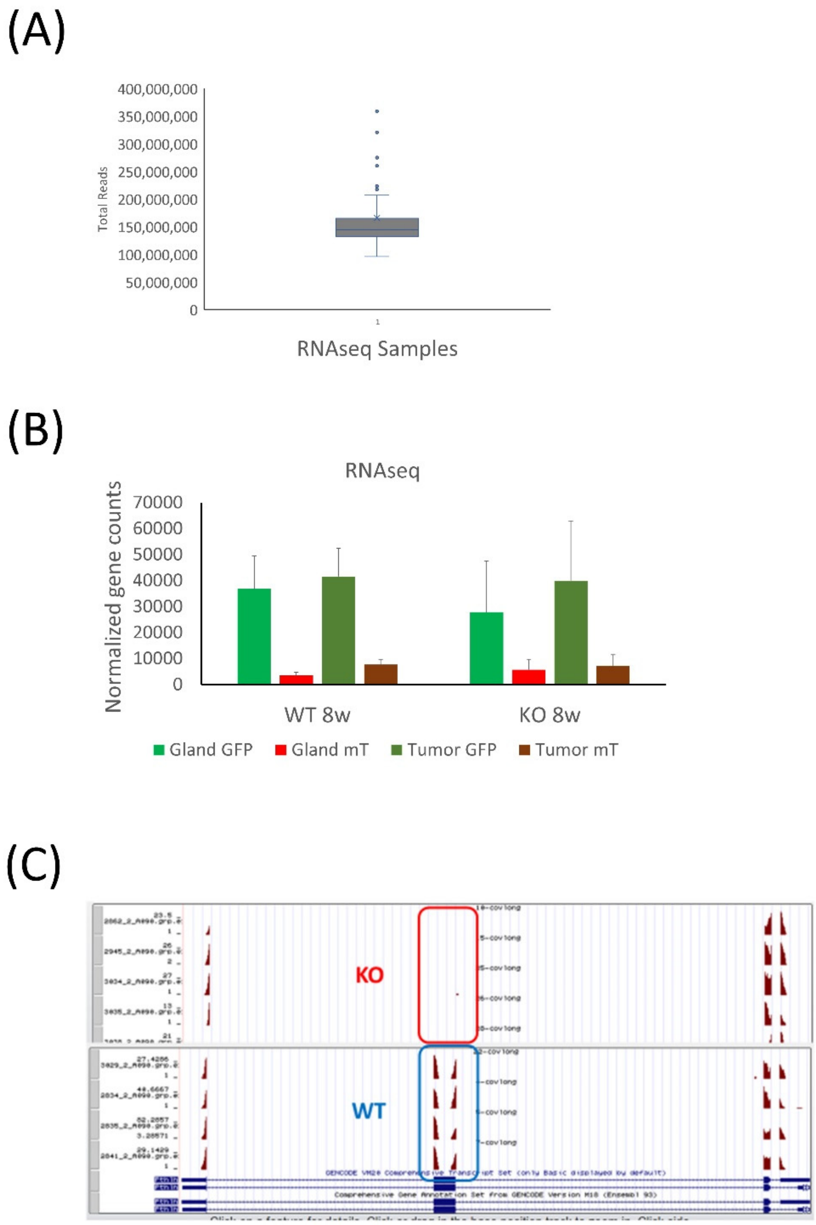

Supplementary Figure S3. (A) Total reads and mapping rate for all the RNAseq samples (n = 33). The box plot presents the distribution of each sample for the total reads and mapping rate for the RNAseq. (B): A representative figure showing the normalized gene counts of GFP and mTd for cancer and control sample at week 8. The green box denotes GFP, and the red box indicates mTd. The normalized gene counts were plotted with the error bar showing the standard deviation. (C): Alignment results of sequencing reads in the *Pthlh* gene (*Pthrp*) cluster are shown as custom tracks in UCSC Genome Browser. The top four tracks show no reads aligned to the exon 4 of *Pthlh* gene. The bottom 4 tracks show exon 4 of *Pthlh* gene is covered by sequencing reads.

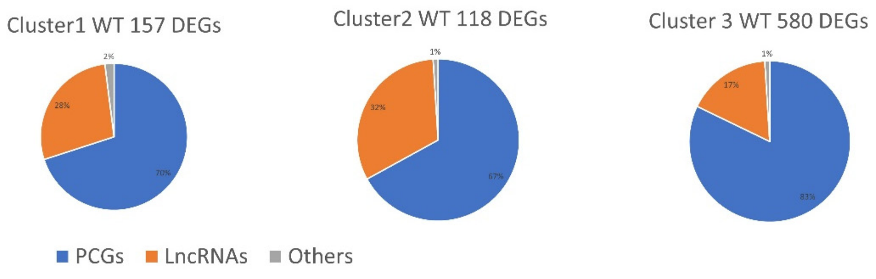

Supplementary Figure S4. The composition of the DEGs from 3 clusters of WT tumors.

(A)

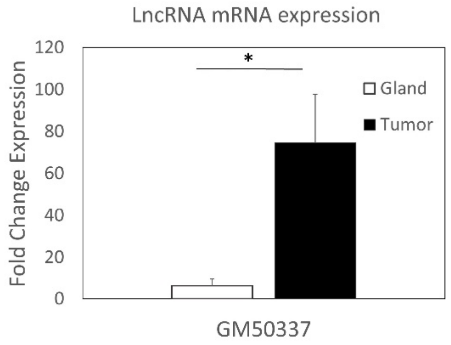

(B)

ID	Label	Coding probability	Peptide length(aa)	Fickett score	Isoelectric point	ORF integrity	Details
Gm50337	noncoding	0.00881623	35	0.36371	6.13507080078	complete	View

Result for species name : mm10 with job ID :1638393851							
Data ID	Sequence Name	RNA Size	ORF Size	Ficket Score	Hexamer Score	Coding Probability	Coding Label
0	GM50337-201	1023	105	0.8008	-0.124157934738	0.028346025798761	no

Supplementary Figure S5. (A) qPCR verification and validation of GM50337 from intact tumor tissues and normal mammary gland in the PyMT mouse; RNA samples were not used for RNAseq. Asterisk "*": *p* Value <0.05. (B) Assessing the protein coding potential of GM50337 using Coding Potential Calculator (CPC), and the predicted open reading frame (ORF) and putative peptide. Protein coding potential analysis of GM50337 RNA transcript using Coding-Potential Assessment Tool (CPAT).

(A)

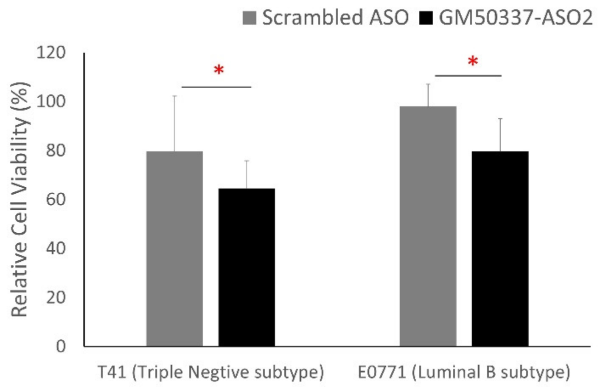

(B)

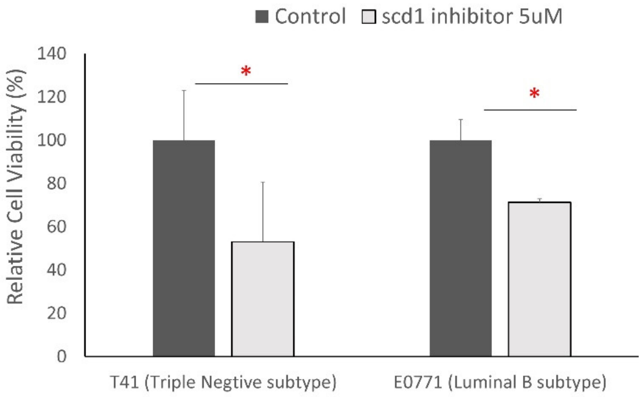

Supplementary Figure S6. (A) mouse T41 and mouse E0771 BC cells treated with 5uM ASO target GM50337 for 72h. A scASO was used as a negative control. Cell viability is normalized to cells treated with scASO. (B) mouse T41 and mouse E0771 BC cells treated with 5uM Scd1 inhibitor for 72 h. Cell viability is normalized to untreated cells (''Control''). Data are presented as mean values ± SD ($n = 3$ independent experiments). *$p < 0.05$ (paired student's t-test; two tailed).

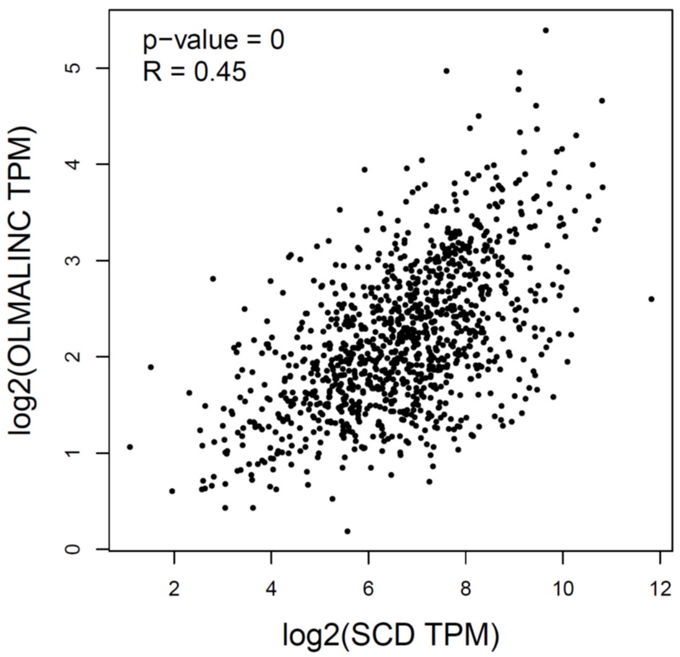

Supplementary Figure S7. Based on the GEPIA2 approach, we obtained the expression correlation between OLMALINC and SCD in TCGA breast cancer dataset.

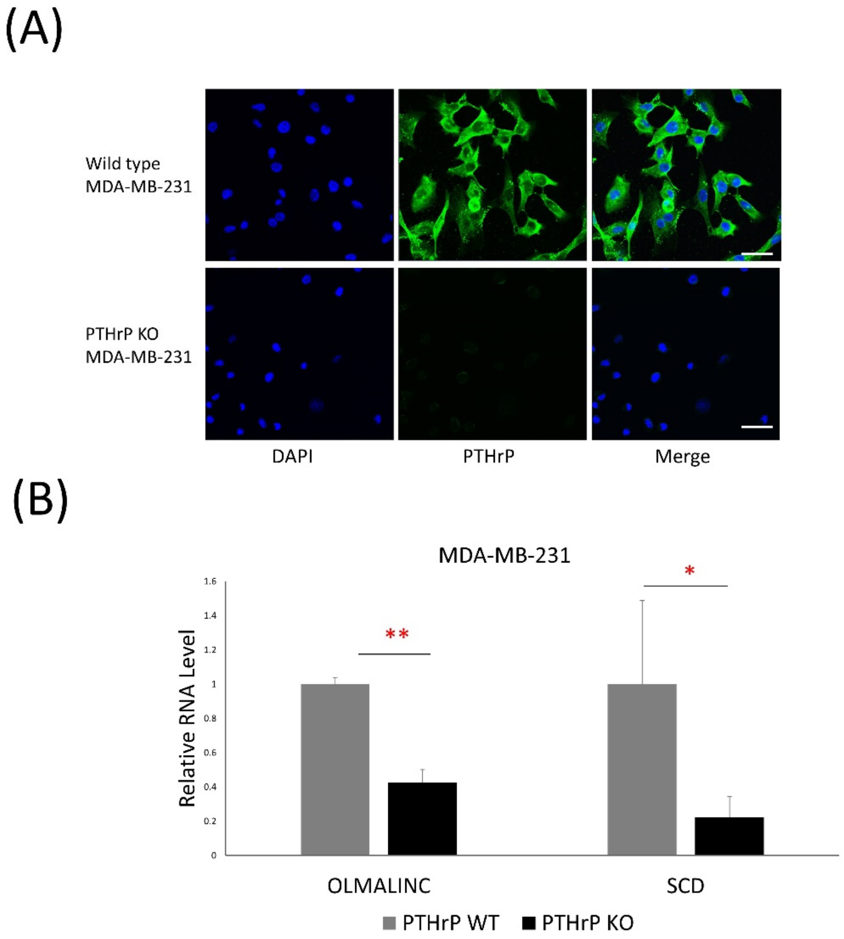

Supplementary Figure S8. (A) Immunofluorescence (IF) for PTHrP in KO or control MDA-MB-231 cell line (empty vector) and fluorescence density diagrams (n = 10, $p < 0.0001$). (B) qPCR showing OLMALINC and SCD expression in MDA231 control and PTHrP KO cells. Data are presented as mean values ± SD (n = 3 independent experiments). *$p < 0.05$, **$p < 0.01$ (paired student's t-test; two tailed).
