# Supplementary material for: PTHrP Regulates Fatty Acid Metabolism via Novel lncRNA in Breast Cancer Initiation and Progression Models
Source: Cancers (Basel). 2023 Jul 25;15(15):3763. doi: 10.3390/cancers15153763 (PMC10417726; doi:10.3390/cancers15153763)
Supplement: Supplementary file 1 [file cancers-15-03763-s001.zip › Table S1.pdf]

Table S1. RNAseq data showing sample name, total reads, mapping rate, duplication rate and gene detected for each sample.

Mouse ID	Total Reads	Gene detected	Mapping rate	Duplication rate
#2971	2.6E+08	27613	0.865	0.44
#3033	2.75E+08	26901	0.764	0.569
#3036	1.52E+08	25028	0.809	0.463
#3039	1.37E+08	23995	0.897	0.349
#3040	1.5E+08	23090	0.809	0.445
#3041	1.33E+08	21755	0.797	0.433
#2944	2.23E+08	24195	0.873	0.501
#3013	1.56E+08	23671	0.848	0.406
#3015	2.07E+08	26948	0.888	0.38
#3034	1.5E+08	26804	0.881	0.352
#3035	1.52E+08	21481	0.698	0.621
#2862	1.34E+08	22746	0.831	0.492
#3038	1.51E+08	23505	0.798	0.478
#2857	1.15E+08	23067	0.831	0.343
#2945	1.32E+08	20852	0.746	0.611
#3002	2.17E+08	28616	0.885	0.421
#3030	1.68E+08	25344	0.877	0.373
#3045	96004162	22228	0.873	0.319
#2880	1.33E+08	24279	0.876	0.344
#2875	1.44E+08	24459	0.87	0.332
#2849	3.21E+08	27808	0.9	0.428
#2781	1.32E+08	24219	0.861	0.344
#2770	1.45E+08	26790	0.879	0.353
#3070	1.42E+08	23722	0.863	0.357
#3048	1.23E+08	24269	0.913	0.348
#3077	1.56E+08	26124	0.898	0.356
#2882	1.26E+08	23628	0.922	0.347
#3065	1.38E+08	26455	0.913	0.337
#2771	3.59E+08	26749	0.939	0.46
#3069	1.07E+08	26558	0.906	0.288
#3026	1.43E+08	26189	0.902	0.325
#3027	1.26E+08	26613	0.909	0.319
